# Intricate Metabolic Network for Paclitaxel Biosynthesis

**DOI:** 10.34133/bdr.0035

**Published:** 2024-05-09

**Authors:** Yuanwei Gou, Xiaojing Jiang, Jiazhang Lian

**Affiliations:** ^1^ Key Laboratory of Biomass Chemical Engineering of Ministry of Education, National Key Laboratory of Biobased Transportation Fuel Technology, College of Chemical and Biological Engineering, Zhejiang University, Hangzhou 310027, China.; ^2^ ZJU-Hangzhou Global Scientific and Technological Innovation Center, Zhejiang University, Hangzhou 310000, China.

## Abstract

Paclitaxel is a renowned broad-spectrum anticancer drug. With the establishment of a chromosome-level high-quality reference genome map of *Taxus*, recent research on paclitaxel biosynthesis has flourished. The oxetane ring is a distinctive chemical moiety of paclitaxel, and three recent studies have proposed different enzymes involved in its formation, reflecting divergent opinions on whether the pathway proceeds via acetylation followed by epoxidation or vice versa. Subsequently, researchers have elucidated gene clusters responsible for the biosynthesis of the key intermediate baccatin III. Despite varying reports, two studies successfully achieved heterologous biosynthesis of baccatin III by transient expression in tobacco. Taxadiene 5α-hydroxylase (T5αH), the first cytochrome P450 in the pathway, exhibited varied product profiles upon heterologous expression systems, contrasting with observations in native *Taxus* species, probably due to differences in partner proteins or cellular microenvironments. Further elucidation of biosynthesis mechanisms, including the reaction order and the promiscuity of key enzymes, is anticipated through collaborative efforts among botanists, chemists, and synthetic biologists.

## Introduction

Discovered in 1964 from the bark of the Pacific yew tree (*Taxus brevifolia*), paclitaxel stands as a US Food and Drug Administration (FDA)-approved anticancer medication (Taxol) utilized in treating breast, lung, prostate, and ovarian cancer [1]. Due to the limited availability in plants, with only about 40 mg found per 1 kg of *Taxus* bark [2], alternative approaches have been developed to isolate precursors (such as baccatin III) from plant cell cultures or *Taxus* tree biomass, followed by chemical conversion into paclitaxel [3]. Nonetheless, with projections of over 28 million cancer cases in the next 20 years [4], semisynthetic strategies still rely on natural resources and artificial cultivation, urging the pursuit of more sustainable production methodologies.

Ideally, metabolic engineering strategies could be employed to elevate paclitaxel levels in *Taxus* cell cultures or to refactor its production in heterologous hosts (such as *Saccharomyces cerevisiae*). Recent successful instances include vinblastine [5,6,7], another anticancer drug with a highly complex biosynthetic pathway involving 31 plant-derived enzymatic steps, which has been de novo biosynthesized in microorganisms. However, the prerequisite for implementing metabolic engineering strategies lies in the comprehensive elucidation of the paclitaxel biosynthetic pathway.

**Fig. 1.**
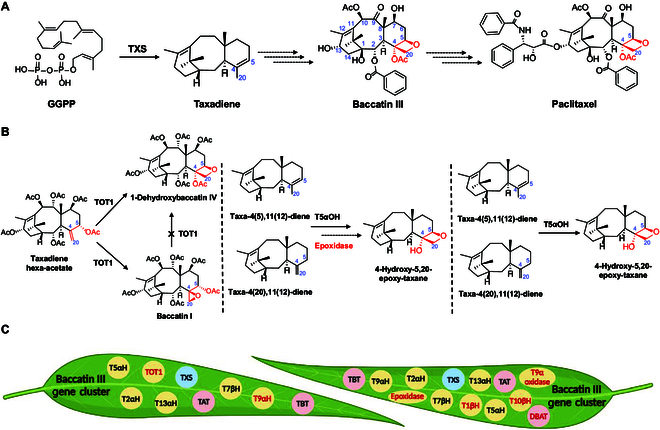
Paclitaxel biosynthetic pathway and divergent findings regarding oxetane ring formation. (A) Abbreviated paclitaxel biosynthetic pathway. (B) Three distinct reports of enzymes involved in oxetane ring formation. The left was proposed by Yan and coworkers, the medium by Fernie and coworkers, and the right by Kampranis and coworkers. (C) Gene clusters responsible for baccatin III biosynthesis. The left was proposed by Yan and coworkers, and the right by Fernie and coworkers. TXS, taxadiene synthase; T5αH, taxane 5α-hydroxylase; T2αH, taxane 2α-hydroxylase; T13αH, taxane 13α-hydroxylase; T7βH, taxane 7β-hydroxylase; T9αH, taxane 9α-hydroxylase; TAT, taxadiene-5α-ol-O-acetyl transferase; TBT, taxane-2α-*O*-benzoyltransferase; TOT1, taxane oxetanase 1; epoxidase, 2-oxoglutarate/Fe(II)-dependent dioxygenase; T10βH, taxane 10β-hydroxylase; T1βH, taxane 1β-hydroxylase; DBAT, 10-deacetyl baccatin III-10-*O*-acetyltransferase.

Over the past two decades, researchers have uncovered approximately 20 enzymatic reactions potentially involved in paclitaxel biosynthesis. These enzymatic steps can be divided into three stages: first, the conversion of the diterpenoid precursor geranylgeranyl diphosphate (GGPP) to form a taxane skeleton with a 4(20)-ene-5α-yl moiety; second, the production of baccatin III through the epoxidation of the C4 and C20 double bonds and successive hydroxylation and acetylation; third, consecutive side-chain modifications at the C2 and C13 positions (Fig. 1A). However, several crucial steps in paclitaxel pathway remains elusive until Yan and coworkers [8] constructed the first chromosome-level high-quality reference genome map of *Taxus*, providing a genomic blueprint and crucial candidate genes. Consequently, progress in paclitaxel biosynthetic pathway has flourished.

## Enzymes Involved in Oxetane Ring Formation

The oxetane ring serves as a distinctive chemical moiety of paclitaxel, playing a crucial role in its biological activity [9]. Recent studies have presented markedly different perspectives on the biosynthesis of this unique chemical structure in nature. Yan and coworkers [10] transiently expressed genes from the CYP725A subfamily in tobacco and injected chemically synthesized substrate taxadiene hexa-acetate to identify oxygenases responsible for forming the oxetane ring. They identified a bifunctional enzyme capable of simultaneously generating 1-dehydroxybaccatin IV with an oxetane ring structure and baccatin I with an epoxide structure, naming it taxane oxetanase 1 (TOT1) (Fig. 1B). By expressing TOT1 in insects to circumvent the interference of endogenous cytochrome P450 enzymes in tobacco and by knocking it down in *Taxus* cells, they further demonstrated that TOT1 mainly catalyzed oxidative rearrangement of the double-bond to form an oxetane ring. Another subsequent study employed semisynthetic 2α-benzoyloxy-7β-acetoxytaxusin as a substrate and screened for a similar enzyme in yeast at almost the same time [11], with similar product profiles including 2-deacetyl-2α-benzoylbaccatin I with an oxetane ring structure and 1β-dehydroxybaccatin VI with an epoxide structure.

Conversely, Fernie and coworkers [12] discovered a 2-oxoglutarate/Fe(II)–dependent dioxygenase (epoxidase), whose transient expression in tobacco, along with taxadiene 5α-hydroxylase (*T5αH*) and precursor pathway genes, resulted in decreased levels of oxidized taxadiene. The epoxidase was demonstrated to catalyze the conversion of taxusin to taxusin-4β,20-epoxide, suggesting its role in epoxidation at the C4 and C20 double bonds (Fig. 1B). Additionally, Kampranis and coworkers [13] reported that the first P450 enzyme in the paclitaxel biosynthetic pathway, CYP725A4 (T5αH), not only catalyzes hydroxylation at the C5 position but also contributes to oxetane ring cyclization (Fig. 1B). The divergent findings concerning oxetane ring formation partly reflect the intricacy of the paclitaxel biosynthetic pathway.

## Minimal Gene Clusters for Baccatin III Biosynthesis

After uncovering enzymes responsible for oxetane ring formation, recent studies have proposed two gene clusters involved in the biosynthesis of the key intermediate baccatin III from taxadiene (Fig. 1C). Both clusters have been transiently expressed in tobacco, resulting in successful detection of baccatin III production. Among these, the cluster with nine genes reported by Yan and coworkers [10], which includes the newly identified TOT1 and T9αH, represents the current minimal gene cluster for baccatin III biosynthesis. In contrast, Fernie and coworkers [12] reported a cluster based on the aforementioned minimal gene cluster, lacking TOT1 but featuring an additional five genes: T10βH, DBAT, as well as their newly identified epoxidase, T1βH, and T9α oxidase. Furthermore, they identified a native *Taxus* β-phenylalanine-CoA ligase, whose co-expression with *Arabidopsis thaliana* benzoate-CoA ligase (BZO), BAPT, 3′-*N*-debenzoyl-2′-deoxytaxol-*N*-benzoyltransferase, and T2′aH in *Nicotiana benthamiana* leaves successfully resulted in the accumulation of paclitaxel, when benzoic acid, _L-_phenylalanine, and baccatin III were externally supplemented. The revelation of gene clusters associated with baccatin III biosynthesis opens avenues for reconstructing the paclitaxel pathway in heterologous hosts, promising to revolutionize current paclitaxel production strategies.

## Varied Product Profile of Heterologously Expressed T5αH

In the reconstruction of the early paclitaxel biosynthetic network, many studies have observed the promiscuous catalytic activity of T5αH, considering it a bottleneck in paclitaxel biosynthesis [13,14]. T5αH exhibits varied product profiles in heterologous expression systems such as *Escherichia coli* [15]*, S. cerevisiae* [16]*,* and *N. benthamiana* [17], resulting in the formation of multiple oxidized taxadiene products. Among these, only a small fraction, such as taxadien-5α-ol, was generally regarded as the productive precursor to paclitaxel, with the rearranged products 5(12)-oxa-3(11)-cyclotaxane (OCT) and 5(11)-oxa-3(11)-cyclotaxane (iso-OCT) being structurally characterized. Attempts to mitigate T5αH over-oxidation in tobacco by substituting the strong constitutive 35S promoter with weaker constitutive promoters have shown partial success, yet undesired mono-oxidized taxadienes like OCT and iso-OCT still account for over 65% of the accumulated taxanes [14]. Notably, the accumulation of OCT, iso-OCT, or related molecules has not been observed in native *Taxus* species [18], possibly due to *Taxus*-specific partner proteins (e.g., metabolon [19]) or microenvironment differences. Future endeavors to address the heterologous expression incompatibility are suggested to enhance the metabolic flux toward taxadien-5α-ol and to mitigate the complex product deconvolution arising from multiple oxidation products [20,21].

## Conclusions

Recent studies have marked a milestone in the field of plant natural product biosynthesis. Although some controversies still surround the paclitaxel pathway, the associated gene clusters have largely been identified. Reconstructing stable paclitaxel biosynthetic cell factories in heterologous hosts promises to revolutionize current production strategies, providing a sustainable and eco-friendly solution to meet the increasing market demands while enhancing the biosynthesis of paclitaxel derivatives with improved pharmacological properties. Moreover, the elucidation of reaction order holds the potential to deepen our understanding of the pathway. As research advances, it becomes evident that the biosynthesis mechanisms are much more intricate than previously expected, featured by the promiscuity of numerous P450s and acetyl or aryl acetyltransferases, exacerbated by the commercial unavailability of most pathway intermediates. These challenges pose obstacles to elucidate reaction order and impede metabolic engineering efforts. The recent strides made in understanding the highly oxygenated tetracyclic core skeleton of paclitaxel stand as a compelling example, underscoring the imperative for collaborative endeavors among botanists, chemists, and synthetic biologists.
